# Conversion Disorder With Mutism as the Presenting Symptom

**DOI:** 10.7759/cureus.90421

**Published:** 2025-08-18

**Authors:** Maria Fernanda Sánchez-Flores, Ana Díaz-Flores, Paula Reyes-Hazim, Nathalia Beras Ramirez, Valeria García-Taveras

**Affiliations:** 1 Psychiatry, Centerstone Behavioral Hospital and Addiction Center, Bradenton, USA; 2 School of Medicine, Faculty of Health Sciences, Pontificia Universidad Católica Madre y Maestra, Santiago, DOM; 3 Psychiatry, Hospital Docente Dr. Francisco E. Moscoso Puello, Santo Domingo, DOM; 4 Public Health and Preventive Medicine, Universidad Central del Este, San Pedro de Macoris, DOM

**Keywords:** antidepressants, antipsychotics, case report, conversion disorder, functional neurological symptom disorder, mutism

## Abstract

Conversion disorder presents as a complex clinical challenge characterized by the manifestation of sensory and motor symptoms without identifiable neurological origins. This case report explores the case of a 20-year-old female with mutism attributed to conversion disorder. Despite extensive medical evaluations ruling out organic causes, the patient exhibited a complete inability to articulate words, attributed to acute conversion disorder with speech symptoms. The treatment approach involved a combination of antipsychotic and antidepressant medications, resulting in symptom resolution within two weeks. However, symptom relapse occurred upon discontinuation of medication, indicating a likelihood of future relapses. Differential diagnoses including neurological diseases, somatic symptom disorder, selective mutism, and factitious disorder were considered and ruled out based on clinical presentation and diagnostic criteria. This case highlights the importance of a thorough multidisciplinary diagnostic approach and individualized treatment strategies, including pharmacotherapy and supportive care, in managing conversion disorder. Recognition of temperamental, environmental, and socioeconomic risk factors may aid early diagnosis and improve prognosis in atypical presentations such as mutism.

## Introduction

Conversion disorder poses a significant challenge in clinical practice due to its variable presentation, diagnostic complexity, and frequent overlap with other neuropsychiatric conditions [1]. It is characterized by the development of sensory or motor symptoms that are inconsistent with recognized neurological or medical conditions and often arise from psychological distress. Reported prevalence in the general population ranges from two to five per 100,000 per year, with higher rates in females and younger adults, though it is likely underdiagnosed due to stigma and misclassification [1]. Conversion disorder can profoundly affect patients' daily functioning and quality of life, requiring individualized and multidisciplinary management strategies [1,2].

Mutism can be defined as: “inability or unwillingness to speak, resulting in an absence or marked paucity of verbal output’’ [3]. Patients with conversion-related mutism typically remain alert, oriented, and cooperative despite their speech impairment. Differential diagnosis is essential to exclude neurological diseases, somatic symptom disorder, selective mutism, and factitious disorder, using thorough clinical evaluation and established diagnostic criteria such as those outlined in the Diagnostic and Statistical Manual of Mental Disorders, Fifth Edition (DSM-5) [3].

The differential diagnosis considers somatic symptom disorder, selective mutism, and factitious disorder. 

The key similarities between conversion disorder and somatic symptom disorder are that symptoms are not intentionally produced and not due to conscious deception, and cause distress/impairment in both disorders. The difference is that somatic symptom disorder is driven by health anxiety. The similarity between conversion disorder and factitious disorder is that both may present with unusual or dramatic neurological symptoms; the difference is that factitious disorder is intentional. Between conversion disorder and selective mutism, both can have onset related to psychological stress, and symptoms are behavioral/functional and not explained by structural disease. However the difference is that selective mutism is driven by anxiety/social inhibition [4].

The diagnostic criteria for conversion disorder with speech symptoms are outlined in the DSM-5; 300.11 (F44.4) [4]: "One or more symptoms of altered voluntary motor or sensory function", "Clinical findings can provide evidence of incompatibility between the symptom and recognized neurological or medical conditions", "Another medical or mental disorder does not better explain the symptom or deficit" and "The symptom or deficit results in clinically significant distress or impairment in social, occupational, or other vital areas of functioning or warrants medical evaluation" [4].

Treatment intervention, which combines antipsychotic and antidepressant medications, can result in symptom resolution within two weeks. Nevertheless, discontinuation of the medication can lead to a relapse, emphasizing the need for further research to optimize therapeutic approaches for this complex disorder [5,6].

This case highlights the critical need for early recognition and effective management of conversion disorder to enhance patient outcomes. Furthermore, it underscores the necessity for personalized treatment plans tailored to the individual needs of patients, as well as the ongoing research required to refine and optimize therapeutic strategies for this condition [1].

## Case presentation

A previously healthy 20-year-old female presents to the psychiatric outpatient consultation with mutism for the past five months. She was sent for psychiatric evaluation after a thorough evaluation of her symptoms by an otolaryngologist and a neurologist with no significant findings or possible causes. Although no speech-language pathologist was available in the center, previous tests (complete blood count (CBC), audiometry, head MRI, video laryngoscopy, and electromyography) were all normal, concluding that there were no vocal cord issues or neurological causes.

The patient presents to the consultation with a well-groomed appearance. She is alert and oriented to person, time, and place. During the interview, the patient was cooperative and attentive. The mood was euthymic, and affection was appropriate. Physical examination to rule out physical causes revealed no abnormalities besides speech dysfunction. The patient was not able to articulate any words but was able to make sounds, if asked questions to express yes or no, and communicate through writing and/or texting. There are no known medical conditions, as well as no previous psychiatric history. The patient is single and currently unemployed, and before the onset of her symptoms, she was attending college studying Education. She lives with her aunt to be near her university. She is an only child and reports a good relationship with her parents but has no current supportive friendships. She denies substance use. Family medical history is unremarkable. She states that after having an upper respiratory illness, she started to lose the ability to articulate words, leading to a complete inability to speak. She did not take any medication; it was self-limited and resolved in three days.

She came to the consultation with her aunt, who has been accompanying her to all previous doctor’s appointments. Her aunt described the patient as a responsible young woman who has never gotten into any trouble and expressed that she admired how the patient handled the situation. The patient underwent a mental status evaluation, which revealed no abnormalities. When the patient was interviewed with her aunt present, she seemed shy and limited the information and details given about the questions asked. Therefore, we decided to interview the patient alone to give her space to fully express herself. No formal psychometric testing was performed; however, a comprehensive interview was conducted. When the patient was interviewed alone, she said that even though other people see her as an easy-going person, she describes herself as very sentimental, empathetic, and easily affected by her surroundings. After being asked if she had any traumatic experiences before the onset of her symptoms, she said that she had lost her grandfather in October 2018, with whom she lived; her boyfriend in August 2018; and a close friend in December 2018, all in unexpected ways. She presented to our consult in April 2022, almost four years after these events. She expressed that since those tragedies occurred, she feels sad about everything that happened but hopeful that she will get better. 

Due to her symptoms and that she met all four diagnostic criteria, previously mentioned, she was diagnosed with acute conversion disorder with speech symptoms. In considering the differential diagnosis for the patient's condition, several possibilities were evaluated and ruled out. Neurological diseases such as aphasia, characterized by language impairment due to brain damage, were excluded based on the patient's ability to understand questions, follow commands, and communicate effectively through writing and texting, coupled with normal MRI results showing no evidence of brain injury. Somatic symptom disorder, marked by excessive worry and abnormal responses to physical symptoms, was also eliminated due to the patient's lack of excessive concern, displaying instead a “la belle indifférence” attitude, which denotes a lack of distress despite medical symptoms. Selective mutism, a disorder where individuals fail to speak in certain situations but can speak in others, was ruled out as the patient demonstrated the inability to communicate in any environment. Lastly, malingering, where patients feign or exaggerate symptoms for personal gain, was discounted as there was no indication that the patient sought anything from medical staff. Factitious disorder was also unlikely, as there is no evidence of intentional symptom production or sick-role-seeking behavior. These considerations collectively helped narrow down and exclude potential differential diagnoses, leading to the diagnosis of acute conversion disorder with speech symptoms. 

The initial treatment plan was aripiprazole 1 mg/ml (oral route) every 12 hours and sertraline 50 mg once a day, with further evaluation two weeks after starting the treatment plan. The decision to treat her as an outpatient was made because she had no suicide risks or any signs that she could be harmful to herself or others. No other treatment was recommended, such as cognitive behavioral therapy (CBT). 

Following the initiation of treatment, the patient presented at the outpatient department two weeks later with a complete resolution of her symptoms. She reported a significant improvement in her ability to speak, noting a specific instance while she was taking a shower where she regained full speech temporarily despite intermittent voice loss. Considering the improvement of her symptoms, the psychiatry team decided to continue her previous medication regimen without any changes in medication or dosage. 

Upon the last follow-up two months later, the patient presented with hoarseness. Despite consistent adherence to treatment, the patient experienced a lapse in aripiprazole supply two weeks prior to the consultation. She noted an exacerbation of symptoms since discontinuation, specifically episodes of voicelessness. Despite symptom aggravation, the patient reported a positive mood and improved mental clarity. When queried about her apprehension of relapse, she rated her fear at a level of four on a scale of one to 10. The treatment approach will remain unchanged, with the addition of ashwagandha 900 mg once daily and Rhodiola 500 mg once daily. The incorporation of adaptogens was determined based on the patient's expressed anxiety, corroborated by her self-identification as predisposed to such feelings and a history of managing them (Table 1). Due to her decline in functioning, she had to discontinue her college studies. This was the patient's last follow-up.

**Table 1:** Timeline of Events

Timeline Event	
Initial presentation	Patient presents with mutism persisting for five months.
	Extensive medical evaluations rule out organic causes.
	Previous tests (Complete Blood Count (CBC), audiometry, head magnetic resonance imaging, video laryngoscopy, electromyography) are normal.
Psychiatric evaluation	Patient referred for psychiatric evaluation.
	Physical examination shows no abnormalities besides speech dysfunction.
	No prior psychiatric diagnoses in patient's medical history.
Diagnosis and Treatment Plan	Diagnosed with acute conversion disorder with speech symptoms (DSM-5 criteria).
	Treatment: aripiprazole and sertraline.
Follow-up Assessments	Two weeks post-treatment: Symptom resolution and improvement in speaking ability reported.
	Two months post-treatment: Hoarseness and symptom relapse due to medication discontinuation. Ashwagandha and Rhodiola were added to the treatment plan.
Ongoing Management	Treatment approach remains unchanged, focusing on medication management and stress resilience.

## Discussion

The case report offers a detailed assessment of a 20-year-old female presenting with mutism as a symptom of conversion disorder. Strengths include its comprehensive evaluation, systematic diagnostic approach, multidisciplinary collaboration, patient-centered care focus, and clear treatment approach using medication. However, limitations include potential lack of generalizability, short follow-up duration, limited discussion on psychological and physical therapy. Future studies focusing on the efficacy of specific therapeutic interventions, long-term outcomes, and factors influencing relapse rates would contribute to a deeper understanding of conversion disorder with mutism. 

Although conversion disorder is infrequent in psychiatric practice, establishing a precise diagnosis is crucial for symptom resolution, as it significantly impacts the patient's quality of life. Consequently, clinicians across specialties must familiarize themselves with its presentation. Instances of conversion disorder, particularly manifesting as mutism, are rare in clinical settings [5]. Multidisciplinary collaboration is essential. This patient was initially assessed by a neurologist, followed by otolaryngology (ENT), to exclude structural or neurologic etiologies such as vocal fold paralysis, mass, or central/peripheral neurologic disease.

In the literature, there is one case described by Thorpe, Keegan, and Veeman [6] where one woman presented with mutism after a traumatic event, which resolved with a combination of CBT and psychodynamic therapy. Similarly, Guimarães and colleagues described a 32-year-old woman with conversion aphonia, normal laryngoscopic findings, and rapid recovery after multidisciplinary evaluation and voice therapy [7]. These cases underscore the importance of being able to recognize conversion disorder.

Per DSM-5 criteria, conversion disorder presents several risk and prognostic factors [8]. Notably, the patient exhibited two such factors: temperamental and environmental. Initially, she self-described as sentimental and easily influenced by her surroundings, indicative of temperamental predisposition. Additionally, the patient experienced multiple traumatic events, namely the loss of three close individuals. Although remote, these events remain salient psychosocial risk factors. Conversion disorder is statistically more prevalent among females and is often associated with low socioeconomic status and rural residency [1]. This patient resides in San Juan de la Maguana, one of the most impoverished regions in the Dominican Republic, underscoring the socioeconomic context of her condition [9].

The patient fulfills diagnostic criteria for functional neurological disorder, evidenced by a prolonged inability to speak without discernible medical cause, significantly impeding daily functioning, including discontinuation of college studies. Notably, her apparent lack of distress despite severe symptoms raises suspicion of la belle indifference [10]. This term is defined as an absence of psychological distress or concern despite the presence of a serious symptom related to a health condition. It is frequently attributed to conversion disorder because it appears in the majority of cases in patients with this condition, but it is not a diagnostic criterion [10].

Although CBT and symptom-specific physical therapy (e.g., speech therapy for mutism) are considered conventional treatments, our approach utilized antipsychotics and selective serotonin reuptake inhibitors (SSRIs) with the aim of improving symptoms [11,12]. While antipsychotics typically offer rapid symptom relief, the addition of SSRIs addresses underlying anxiety, highlighting the multifaceted nature of treatment [13]. Specifically, ashwagandha and Rhodiola were used, both of which have been associated with reductions in stress-related conditions and anxiety [11,14,15]. Although early diagnosis portends a more favorable prognosis, the patient had a rapid recovery despite having the symptoms for five months. Furthermore, her symptom relapse upon medication cessation suggests a propensity for future recurrences, leading to explore the treatment time frame for conversion disorder [15].

## Conclusions

This case report highlights the importance of a comprehensive evaluation and a multidisciplinary approach in diagnosing and managing conversion disorder with mutism. The combination of a systematic diagnostic process, a well-defined treatment strategy, and interdisciplinary collaboration was key to the successful management of the patient. Early recognition of conversion disorder is essential to improving quality of life, particularly in atypical presentations such as mutism, where it should be considered once organic causes have been excluded.

Risk factors, including temperamental predisposition and adverse environmental events, along with a vulnerable socioeconomic background, may play a significant role in its development. Despite the absence of a standardized treatment, the patient’s positive response to antipsychotics and antidepressants underscores the need to explore alternative therapeutic approaches beyond CBT.
